# Antibacterial Activity of 2-Hydroxyisocaproic Acid (HICA) Against Obligate Anaerobic Bacterial Species Associated With Periodontal Disease

**DOI:** 10.1177/11786361211050086

**Published:** 2021-10-21

**Authors:** Marjut Sakko, Riina Rautemaa-Richardson, Samuli Sakko, Malcolm Richardson, Timo Sorsa

**Affiliations:** 1 Department of Oral and Maxillofacial Diseases, Clinicum, University of Helsinki, and Helsinki University Hospital, Helsinki, Finland; 2 Division of Infection, Inflammation and Respiratory Medicine, Faculty of Biology, Medicine and Health, The University of Manchester, Manchester, UK; 3 Department of Infectious Diseases, Manchester Academic Health Science Centre, Wythenshawe Hospital, Manchester University NHS Foundation Trust, Manchester, UK; 4 Mycology Reference Centre Manchester, ECMM Excellence Centre of Medical Mycology, Manchester University NHS Foundation Trust, Manchester, UK; 5 Manchester Fungal Infection Group, Division of Infection, Immunity and Respiratory Medicine, University of Manchester, Manchester, UK; 6 Department of Oral Diseases, Karolinska Institutet, Huddinge, Sweden

**Keywords:** Anaerobic bacteria, bactericidal, 2-hydroxyisocaproic acid, 2-hydroxy-4-methyl pentanoic acid, 2-hydroxy-4-methyl valeric acid, leucic acid, new topical antimicrobial

## Abstract

Topical antiseptics are used to assist and further increase the effect of mechanical biofilm eradication and to potentially prevent new biofilm formation in periodontal treatment. This is of importance in treatment-resistant infections with 10% prevalence of all periodontitis cases to avoid the need for antibiotic therapy. The purpose of this study was to evaluate the antimicrobial activity of DL-2-hydroxyisocaproic acid on human pathogenic obligate anaerobic bacteria related to periodontitis. In this study antimicrobial activity of 2-hydroxyisocaproic acid was observed against 14 bacterial reference strains and clinical isolates of obligate anaerobic bacterial species using a microdilution method in 1.25 to 160 mg/mL concentrations of 2-hydroxyisocaproic acid. The 11 strains of bacteria included in this study are typically associated with periodontal disease; *Porphyromonas gingivalis*, *Fusobacterium nucleatum*, *Tannerella forsythia*, *Aggregatibacter actinomycetemcomitans*, and *Parvimonas micra*. Three strains of *Cutibacterium acnes*, normally associated with skin diseases, were tested for comparison. 90% inhibitory concentration was determined at 48 hours and minimum bactericidal concentration was determined after 72 hours incubation. The 2-hydroxyisocaproic acid was bactericidal at ⩾160 mg/mL for all isolates tested. The reference strain of *T. forsythia*, and the reference strain and the clinical isolates of C. acnes were the most tolerant ones. The reference strains and clinical isolates of *F. nucleatum* and *A. actinomycetemcomitans* were killed at ⩾40 mg/mL concentration. In conclusion, topical use of 2-hydroxyisocaproic acid could eventually be a well-tolerated and useful method in the therapy of patients with difficult-to-treat periodontal disease or other superficial infections to avoid unnecessary antibiotic use and the emergence of antibiotic resistance.

## Introduction

Periodontitis is an inflammatory disease of periodontal tissues, which is induced by dysbiotic polymicrobial biofilms.^1^ The degree of inflammation and symptoms depends on the quantity of microbial cells and their virulence as well as host responses. Various cell surface proteins and carbohydrates, toxins, and hydrolytic enzymes are considered to be microbial virulence factors because they may by enhancing microbial attachment and protection from the host induce the microbial burden. The ability of microbes to form especially the dysbiotic biofilm is potentially an additional virulence factor.^2^ The ability to find synergistic microbial species, the ability to evade or resist host responses and to release bacterial mediators such as lipopolysaccharides (LPS), and the release of tissue damaging proteolytic enzymes have an impact on microbial survival in multispecies infections.^3^

Bacterial species associated with severe periodontitis are mainly obligate anaerobic rods, such as *Porphyromonas gingivalis*, *Fusobacterium nucleatum*, *Aggregatibacter actinomycetemcomitans*, *Prevotella* spp., *Campylobacter rectus* or some cocci such as *Parvimonas* spp. They are able to live and multiply in the anaerobic areas such as the gingival sulcus. *Treponemas* and viruses can also be typically found in periodontitis. It has been suggested that in the polymicrobial synergy and especially the dysbiosis theory of the pathogenesis of periodontitis some corner-stone pathogens have increased ability to form bacterial communities found in the presence of periodontal disease. Accordingly, commensal microbial species are suppressed.^1^

Topical antimicrobials potentially increase the effect of mechanical biofilm eradication. They may also prevent new biofilm formation as a part of the anti-infective treatment of periodontitis.^4,5^ Antiseptics can be used as an oral rinse by the patient or injected into the gingival sulcus by a dentist. The use of these agents as an adjuvant to scaling and root planning has been suggested to potentially improve the outcome of chronic periodontitis.^6,7^ This improved efficacy would be particularly helpful in the management of treatment-resistant infections representing some 10% of all periodontitis cases in order to reduce the use of systemic antibiotics in this patient group.^8^ Overall, topical antiseptics are potential devices in the treatment of any kind of superficial infections for avoiding the potential spreading of infection.^9^

Hietala et al^10^ identified 2-hydroxyisocaproic acid (HICA) as a bacterial amino acid with antimicrobial potential from a fermentation product of *Lactobacillus plantarum*. It is a by-product of the leucine-acetyl-CoA pathway in the leucine degradation pathway. For HICA elimination hydroxyisocaproic acid dehydrogenase (HicDH) enzyme is needed. Both of them are mainly produced by lactobacilli.^11-23^ The reduction reaction of 2-ketoisocaproic acid (KICA) to HICA is catalyzed by hydroxyisocaproic acid dehydrogenase (HicDH). An NADH-dependent oxygenation reaction is needed for HICA elimination back to KICA.^22^ The broad-spectrum antimicrobial activity of HICA has been shown against facultative anaerobic gram-positive and gram-negative bacteria,^10,24^ obligate anaerobic *F. nucleatum*,^24^ several fungal species,^10,25-27^ and against *Candida albicans* biofilms.^28^ HICA exerts anti-inflammatory properties as well.^29^

The European Food Safety Authority (EFSA)^30^ has approved HICA as a flavoring substance in nutritional products. The maximum survey-delivered daily intake is 0.0012 μg/capita/day and theoretical maximum daily intake is 3800 μg/capita/day. The dose of the threshold of concern is 1800 μg/capita/day. HICA is also found to be present as a natural compound in human breast milk in minimal amounts, 17 μg/L.^31^ Selis et al^32^ showed that DL-HICA is not cytotoxic or genotoxic for human periodontal fibroblasts at <10 mg/mL after 24 hours incubation. They considered that HICA could be tested as a root canal medication for regenerative endodontics. Cytotoxicity of the widely used antiseptic chlorhexidine on human cells is well-reported in the literature. Verma et al^33^ showed that chlorhexidine was toxic to periodontal fibroblasts beyond 10 mg/mL even after 1, 5, and 15 minutes incubation. Since HICA can be metabolized by human tissues^34,35^ and is found naturally in nutrition it should be well-tolerated at least in the doses recommended by EFSA. HICA could eventually be a useful new and safe topical antimicrobial agent.

The hypothesis of this study was that HICA could be active also against obligate anaerobic periodontopathogenic bacteria, because it affects the microbial metabolism. The purpose of this study was to evaluate the microbial activity and spectrum of HICA against obligately anaerobic bacterial in an environment that is favorable for their growth.

## Materials and Methods

### Bacterial strains

Fourteen obligate anaerobic strains were included in the study: 7 reference strains and 7 clinical isolates of *Porphyromonas gingivalis*, *Fusobacterium nucleatum*, *Tannerella forsythia*, *Aggregatibacter actinomycetemcomitans*, *Parvimonas micra*, and *Cutibacterium acnes*. Nine isolates belonged to the group of gram-negative rods, 2 of them were gram-positive cocci and 3 others were gram-positive rods (Table 1). In addition to gingival micro-organisms 3 strains of *C. acnes*, which can be found often in skin diseases acted as a control. Clinical isolates were obtained from the Clinical Microbiology Laboratory of Helsinki University Central Hospital HUSLAB (PD and TS isolates; Finland), and from the Department of Oral and Maxillofacial diseases, University of Helsinki (A, T, and R isolates; Finland). The reference strains were obtained from the American Type Culture Collection (ATCC; USA). The bacterial strains and isolates were identified by standard biochemical methods.

**Table 1.** Obligate anaerobic bacterial strains included in the study (n = 14) are listed in this table. Their designations, sources, minimum bactericidal concentrations (MBC) of HICA at 72 hours are shown.

Organism	Designation	Source	MBC at 72 h (mg/mL)
Gram-negative rods			
*Porphyromonas gingivalis*	ATCC 33277	Gingival sulcus	⩾80
*Porphyromonas gingivalis*	T 75048	Gingival sulcus	⩾20
*Fusobacterium nucleatum*	ATCC 25586	Cervico-facial lesion	⩾40
*Fusobacterium nucleatum*	PD 788	Gingival sulcus	⩾40
*Tannerella forsythia*	ATCC 43037	Gingival sulcus	⩾160
*Tannerella forsythia*	R 1015	Oral abscesses	⩾80
*Aggregatibacter actinomycetemcomitans*, *serotype a*	ATCC 29523	Blood	⩾10
*Aggregatibacter actinomycetemcomitans*, *serotype b*	ATCC 43718	Gingival sulcus	⩾40
*Aggregatibacter actinomycetemcomitans*, *non-serotyped*	R 58	Oral abscesses	⩾10
Gram-positive cocci			
*Parvimonas micra*	ATCC 33270	Purulent pleurisy	⩾80
*Parvimonas micra*	A 299	Gingival sulcus	⩾80
Gram-positive rods			
*Cutibacterium acnes*	ATCC 6919	Facial acne	⩾160
*Cutibacterium acnes*	TS 9498	Breast skin	⩾160
*Cutibacterium acnes*	TS 9512	Subarachnoidal lesion	⩾160

Clinical bacterial isolates were obtained from the Clinical Microbiology Laboratory Helsinki University Central Hospital HUSLAB (PD and TS), and from the Department of Oral and Maxillofacial diseases, University of Helsinki (A, R and T). The reference strains were obtained from the American Type Culture Collection (ATCC; USA).

### Antimicrobial susceptibility testing

The susceptibility of obligate anaerobic bacteria for DL-HICA was tested by a modification of the standard microdilution method of the Clinical and Laboratory Standards Institute (CLSI)^36^; Methods for antimicrobial susceptibility testing of anaerobic bacteria, document M11–A5. Culture medium, inoculum concentration, and incubation volume were used according to the manual instructions. Bacterial cell densities were prepared at a concentration of 10^7^ cfu/mL in broth, estimated by using McFarland standard 3.0 and diluted in serial 1:100. This was confirmed also by quantitative plate counts. 90% inhibitory concentration (IC_⩾90_) was determined at 48 hours, which is the MIC (minimum inhibitory concentration) breakpoint according to manual instructions of the CLSI. The minimum bactericidal concentration (MBC) was determined after 72 hours incubation. The concentrations of HICA and the test pH of 6.5 were selected following on from a series of the pilot studies. Increasing concentrations of 1.38, 2.75, 5.5, 11, 22, 44, 88, and 176 mg/mL of HICA were prepared in thioglycollate broth. Controls free of antimicrobial agents were included in all experiments (negative control). Chlorhexidine digluconate (pH 6.5; 0.6 mg/mL) was incorporated as a positive control. To check for sterility, pure thioglycollate broth without bacteria was incubated. Microbial suspensions (20 µL) were added to the test solutions (180 µL) in 96-well cell-culture microtiter plates (F96 MicroWell Plates, NUNC, ThermoFisher Scientific, Leicestershire, UK) resulting in a final bacterial density of 10^6^ cfu/mL in the wells. Final test concentrations of HICA resulted in 1.25 to 160 mg/mL in the wells of the microtiter plates. Bacterial cultures were grown in an anaerobic atmosphere at 35°C for up to 72 hours. Oxygen was eliminated from the anaerobic jars with the Anoxomat^®^ III Anaerobic Culture System (Advanced Instruments, Norwood, Massachusetts, USA). Anaerobic indicators were used to monitor the anaerobic test conditions during incubation (Resazurin Anaerobic Indicator, Oxoid, ThermoFisher Scientific, Leicestershire, UK). The suspensions in the wells were mixed using a sterile instrument to ensure homogenous cultures before measurement. To determine the optical density at 0, 24, 48, and 72 hours a spectrophotometer (450 nm; Victor X4, 2030 Multilabel reader, PerkinElmer, Massachusetts, USA) was used. Then, the microbicidal activity of HICA and the controls were determined by culture on Brucella Blood agar (BD BBL™ Brucella agar with 5% sheep blood, hemin and vitamin K1 prepared media, Anaerobe Systems, ThermoFisher Scientific, Leicestershire, UK) after the last measurement with spectrophotometer. About 20 µL of test suspension from each well was cultured on agar for another 72 hours. The experiments were performed twice in quadruplicate.

### Analysis of bacterial growth

Bacterial growth was determined in each test solution by changes in optical density. Bacterial growth inhibition was determined in comparison to maximum bacterial growth observed in the negative control for each bacterial strain. The results of the spectrophotometrical analyses are presented as means and standard error of mean. The results of bacterial growth inhibition over 90% (IC_⩾90_) for each reference strain and clinical isolate were read at 48 hours. The results of microbicidal activity which were determined by test solution cultures on agar are shown as minimum bactericidal concentration (MBC) at 72 hours.

## Results

HICA was bactericidal at ⩾160 mg/mL for all reference strains (ATCC) and clinical isolates included in the study, at 72 hours (Table 1). The reference strain and the clinical isolate of *P. gingivalis* and *P. micra*, and the clinical isolate of *T. forsythia* were killed at ⩾80 mg/mL HICA. A concentration of ⩾40 mg/mL HICA was bactericidal for the reference strain and the clinical isolate of *F. nucleatum*, and 2 reference strains of *A. actinomycetemcomitans* including serotype a and b and 1 non-serotyped clinical isolate, at 72 hours.

**Figure 1.**
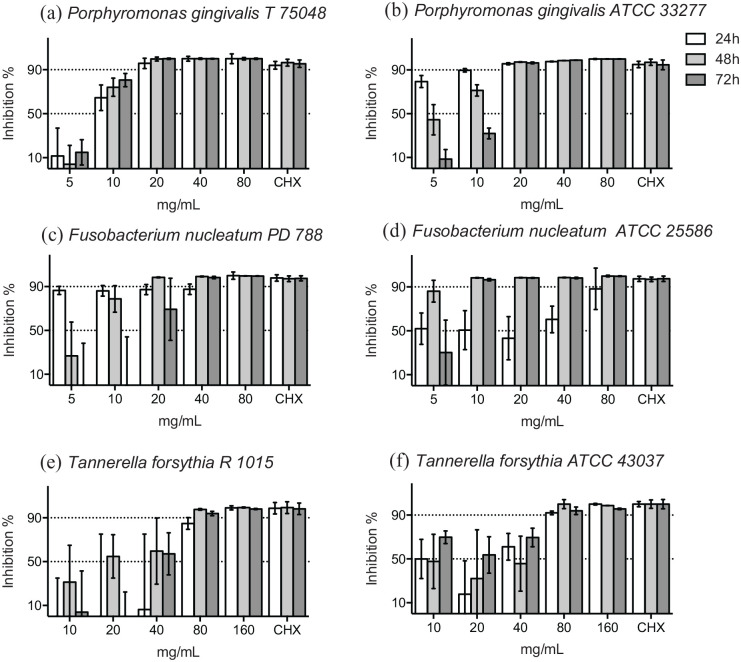
Growth inhibition of gram-negative rods in the presence of increasing concentration of HICA. The activity of 5 to 80 mg/mL of HICA in thioglycollate broth on the clinical isolates (a, c, and e) and the reference strains (b, d, and f) of *P. gingivalis*, *F. nucleatum* and 10 to 160 mg/mL of HICA on *T. forsythia* in anaerobic test conditions at pH 6.5 up to 72 hours is shown. Chlorhexidine digluconate 0.6 mg/mL was used as a positive control and thioglycollate broth as a negative control.

At 48 hours, greater than 90% of bacterial growth of the reference strain, the clinical isolate of *P. gingivalis* and the clinical isolate of *F. nucleatum* were inhibited by ⩾20 mg/mL concentration of HICA (Figure 1a–c). However, over 90% of bacterial growth of the reference strain of *F. nucleatum* was inhibited even by ⩾10 mg/mL concentration (Figure 1d). Bacterial growth inhibition for the reference strain and the clinical isolate of *T. forsythia* was over 90% in the presence of ⩾80 mg/mL HICA at 48 hours (Figure 1e and f). Dose-dependent inhibition was shown by HICA for all reference strains and clinical isolates of *P. gingivalis*, *F. nucleatum*, and *T. forsythia*.

**Figure 2.**
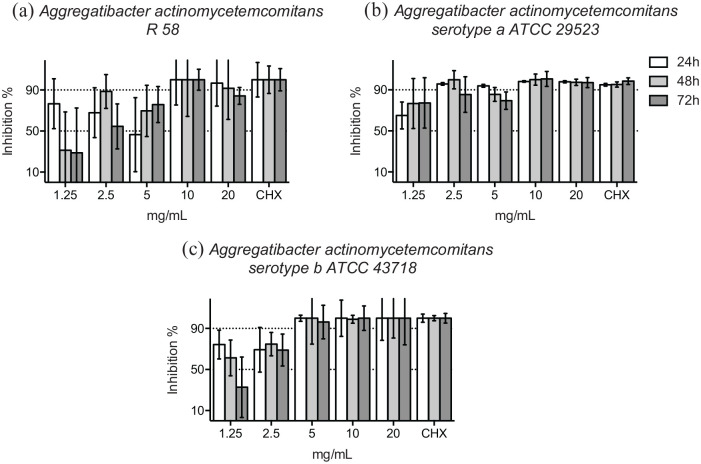
Growth inhibition of gram-negative rods in 1.25 to 20 mg/mL of HICA. The activity of increasing concentrations of HICA in thioglycollate broth on the clinical isolate of *A. actinomycetemcomitans* non-serotyped (a), and the reference strains of serotype a (b) and serotype b (c) at pH 6.5 up to 72 hours in anaerobiosis is shown. Chlorhexidine digluconate 0.6 mg/mL was used as a positive control and thioglycollate broth as a negative control.

Over 90% of the bacterial growth of the non-serotyped clinical isolate and the serotype a reference strain (ATCC 29523) of *A. actinomycetemcomitans* was inhibited in ⩾10 mg/mL concentration of HICA at 48 hours (Figure 2a and b). Over 90% of the growth of the reference strain of *A. actinomycetemcomitans* serotype b (ATCC 43718) was inhibited even in the presence of ⩾5 mg/mL of HICA (Figure 2c). The concentration, in which over 50% but under 90% of the growth of the *A. actinomycetemcomitans* strains was variable, but dose-dependent inhibition could be seen up to 48 hours.

**Figure 3.**
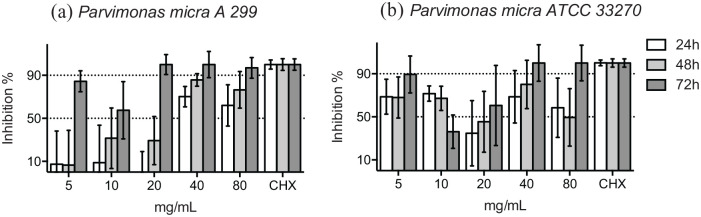
Growth inhibition of gram-positive cocci in 5 to 80 mg/mL of HICA. The activity of increasing concentrations of HICA in thioglycollate broth on the clinical isolate (a) and the reference strain (b) of *P. micra* in anaerobic test conditions at pH 6.5 up to 72 hours is shown. Chlorhexidine digluconate 0.6 mg/mL was used as a positive control and thioglycollate broth as a negative control.

At 48 hours, the growth inhibition of the reference strain and the clinical isolate of *P. micra* was evident in the presence of ⩾40 mg/mL HICA around 50% or higher (Figure 3a and b). Incubation for 72 hours was required to demonstrate 90% growth inhibition in the presence of ⩾40 mg/mL HICA. The bacterial growth inhibition caused by HICA was mainly dose-dependent and especially time-dependent for the clinical isolate of *P. micra* up to 48 hours. The inhibition results of the reference strain were more variable.

**Figure 4.**
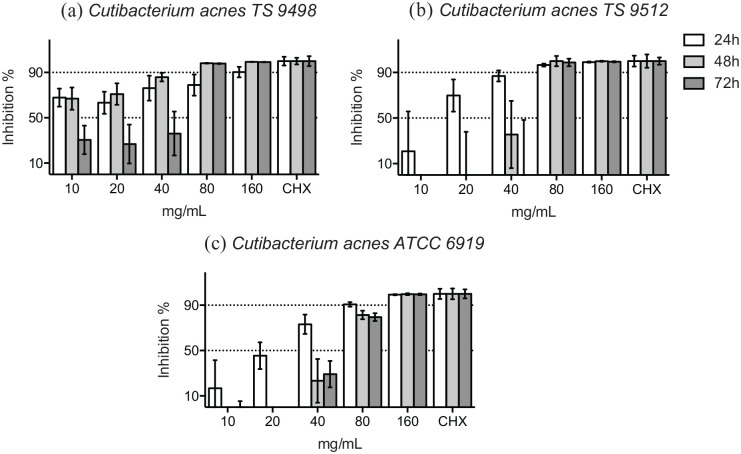
Growth inhibition of gram-positive rods in 10 to 160 mg/mL of HICA. The activity of increasing concentrations of HICA in thioglycollate broth on 2 clinical isolates (a and b) and the reference strain (c) of *C. acnes* at pH 6.5 in anaerobiosis up to 72 hours is shown. Chlorhexidine digluconate 0.6 mg/mL was used as a positive control and thioglycollate broth as a negative control.

*C. acnes* (formerly *Propionibacterium acnes*), was tested as a control in this study. At 48 hours, over 90% of the bacterial growth of 2 clinical isolates of *C. acnes* was inhibited by ⩾80 mg/mL of HICA (Figure 4a and b), but the growth of the reference strain was markedly inhibited by one dilution higher concentration ⩾160 mg/mL of HICA (Figure 4c). Growth inhibition in the presence of HICA was dose-dependent for 2 reference strains and the clinical isolate of *C. acnes* tested at 48 hours.

## Discussion

The main finding of this study was that HICA showed broad-spectrum antibacterial activity against obligate anaerobic bacteria. All bacterial strains and isolates included were susceptible to HICA at a concentration ⩾160 mg/mL. At 48 hours, the IC_⩾90_ results were 1 to 3 dilutions lower for each isolate compared to the MBC at 72 hours. Growth of the reference strain and the clinical isolate of *P. gingivalis* in the negative control was optimal and the growth of the reference strain and the clinical isolate of the *P. micra* was the slightest in this study. HICA was bactericidal against species associated with periodontal disease. In addition, 1 reference strain and 2 clinical isolates of *C. acnes*, which constituted as comparators were killed in the highest test concentration of HICA.

At 72 hours, the MBC of HICA was ⩾80 mg/mL for the reference strain and the clinical isolate of *P. micra*, for the reference strain of *P. gingivalis*, and for the clinical isolate of *T. forsythia*. However, the clinical isolate of *P. gingivalis* was susceptible for HICA at ⩾20 mg/mL concentration. The MBC of HICA was ⩾40 mg/mL for the reference strain and the clinical isolate of *F. nucleatum* and for the b serotype reference strain of *A. actinomycetemcomitans*. The reference strain of *A. actinomycetemcomitans* serotype a and the non-serotyped clinical isolate were the most sensitive to HICA, exhibiting an MBC in ⩾10 mg/mL, which was even a 4-fold lower dilution than with the most tolerant reference strains and clinical isolates in this study. In general, gram-positive bacteria showed to be more resistant to HICA than gram-negative bacteria. This may be due to the differences between their cell wall structures. It is possible that HICA may permeate through the cell wall of gram-negative rods more easily than the cell wall with thick peptidoglycan layer of gram-positive bacteria under the test conditions used here.

In comparison to the results of earlier studies the MBC results for the reference strain and the clinical isolate of *F. nucleatum* were similar. The MBC for *F. nucleatum* was earlier shown to be 4.5 mg/mL in pH 5.2.^24^ In the present study, they were probably killed in 9 times higher concentration because the growth conditions were more favorable at pH 6.5. The activity of HICA against other obligate anaerobic bacterial species included in this study were tested for the first time. The experimental design showed that the growth of all bacterial strains tested is inhibited and prevented by HICA. This is not necessarily enough evidence to prove its efficacy in clinical conditions. However, the results are promising because HICA, which is well-tolerated and metabolized by human cells^34,35^ might have a potential effect on the dysbiotic polymicrobial biofilm, virulence factors of the corner-stone bacterial species or host responses in periodontitis because of its broad-spectrum antimicrobial activity,^10,24-27,37^ the inhibitory activity against microbial biofilms,^28^ and proteolytic enzyme activities.^29^

In a clinical study, in which the effect of oral prophylaxis before root planning and scaling was monitored, it showed no improvements in the treatment outcomes.^38^ If the living circumstances of dysbiotic microflora is worsened by mechanical treatment and could be aided by a safe antibacterial agent, commensal bacteria may have a better chance to recolonize previously anaerobic oral niches. This is a prerequisite for balancing oral cavity microbiome and healing of periodontitis. New and safe topical antimicrobial agents are needed for the prevention and treatment of superficial and dysbiotic infections to avoid potential adverse effects of presently used antiseptics^33^ and antibiotics,^8^ as well as the emergence of antibiotic resistance.^39^ HICA may prove to be a useful agent in the treatment and prevention of topical infections such as moderately advanced and difficult-to-treat periodontal infections.

This study met the objectives of the study and answered the study question as to whether HICA is active against a range of gram-positive and gram-negative obligately anaerobic bacterial species associated with periodontitis. Clinical studies about the potential efficacy of HICA together with root planning and scaling in the treatment of periodontitis are needed in the future.
